# Effect of Team Composition on Integrated Care Within Multidisciplinary Family Doctor Teams in China: Inter-Professional Collaboration as Mediator

**DOI:** 10.5334/ijic.9837

**Published:** 2026-07-01

**Authors:** Yanli Shi, Ganquan Yu, Huilan Zhou, Yushan Ke, Jindi Wen, Xin Wang

**Affiliations:** 1 School of Public Health, Sun Yat-Sen University, Guangzhou, China; 2 Futian District Center for Disease Control and Prevention, Shenzhen, China; 3 Zhengguo Town Health Center, Zengcheng District, Guangzhou, China

**Keywords:** multidisciplinary, family doctor teams, inter-professional collaboration, integrated care, mediation analysis

## Abstract

**Introduction:**

Integrated care (IC) is pivotal to China’s primary healthcare reform under Healthy China 2030. Multidisciplinary family doctor teams (FDTs), with variable compositions, aim to deliver IC but face performance inconsistencies. While prior studies associate team composition with clinical outcomes, they neglect to elucidate the underlying mediating mechanisms. This study examines the association between FDT composition and IC, and whether inter-professional collaboration (IPC) may act as a mediator in this relationship.

**Methods:**

This cross-sectional study surveyed 224 stratified-randomly sampled FDTs in Guangzhou in August 2023. Validated instruments were used to measure IPC and IC. We evaluated the potential mediating role of IPC using bias-corrected bootstrap procedures, and conducted a path analysis to explore the associations between IPC and IC.

**Results:**

For standard teams compared with basic teams, the total effect on IC was not significant (B = 1.986, 95% CI: –0.719 to 4.691). The indirect effect via IPC was significant (B = 1.743, 95% CI: 0.490 to 3.128), while the direct effect was not significant (B = 0.243, 95% CI: –2.157 to 2.643). For advanced teams, the total effect was significant (B = 7.386, 95% CI: 4.316 to 10.456). Both the direct effect (B = 5.351, 95% CI: 2.624 to 8.077) and the indirect effect via IPC (B = 2.035, 95% CI: 0.548 to 3.492) were significant. Path analysis further examined the structural associations between IPC and the multifactorial components of IC.

**Conclusion:**

For advanced teams, team composition was positively associated with IC, with IPC partially mediating this relationship. For standard teams, only the indirect effect via IPC was significant.

## Introduction

According to the latest data released by the World Health Organization, noncommunicable diseases caused the deaths of at least 43 million people in 2021, equivalent to 75% of non-pandemic-related deaths globally [1]. The rising prevalence of chronic conditions and multimorbidity highlights that populations requiring complex healthcare are expanding, straining health systems globally. To address these complexities, integrated care (IC) has emerged as a strategic paradigm, advancing person-centered health systems through coordinated service delivery by multidisciplinary teams operating across care settings and levels of care [2,3].

The family doctor contract system has emerged as a cornerstone of China’s IC implementation, operationalizing the national strategy to strengthen primary care coordination under the Healthy China initiative [4]. Family doctor is responsible for establishing a long-term and stable relationship with their patients. They provide IC to help patients obtain coordinated healthcare. Recent policy initiatives in China have prioritized the institutionalization of family doctor contract services, with measures such as diversifying workforce recruitment channels [5]. Policy-mandated FDTs maintain a core composition of general practitioners, nurses, and public health physicians [6], with flexible configuration frameworks allowing context-specific supplementation by specialists, traditional Chinese medicine (TCM) practitioners, village doctors or non-medical community volunteers to address localized service gaps [7].

A robust implementation of family doctor contract system demonstrates consistent associations with improved health outcomes and cost-effectiveness [8,9]. Therefore, it is important to identify strategies to optimize FDTs’ service quality and operational efficiency. Existing studies have established associations between team composition and healthcare outcomes. Nevertheless, inconsistencies persist across studies, potentially attributed to heterogeneity in study design, sampling frameworks, and outcome variables. For instance, Kristen revealed that multidisciplinary team composition in primary care settings significantly correlated with enhanced patient activation scores [10]. In contrast, Innocent’s study revealed paradoxically lower team functioning scores in teams incorporating pharmacists or social workers alongside family doctors and nurses, compared to teams incorporating family doctors and nurses [11]. These studies have provided valuable insights into how team composition affects healthcare. However, they neglect to elucidate the underlying mediating mechanisms.

Inter-professional collaboration (IPC) refers to the collaboration among healthcare providers from different professional backgrounds aimed at improving the quality of healthcare [12]. Studies have demonstrated that IPC acts as a critical enabler for the implementation of IC [13]. For instance, Campagna et al. highlighted that IPC is a foundational best practice for IC, enabling continuity across the entire care continuum by aligning diverse professional expertise [14]. Therefore, understanding the level of IPC within teams and identifying its dynamic processes is essential for exploring how it mediates the effects on IC. To provide a theoretical foundation for conceptualizing and measuring IPC, this study draws on D’Amour model of IPC [15]. This framework conceptualizes IPC across four core dimensions, namely shared goals and visions, internalization, formalization, and governance.

Therefore, this study conducts a survey of FDTs to address three research questions: (1) whether different compositions of FDTs are associated with the level of IC; (2) whether IPC statistically mediates the association between team composition and IC; (3) if mediation is observed, what is the potential pathway through which IPC is associated with IC?

## Methods

### Study setting and participants

Surveys of FDTs were conducted in Zengcheng District and Panyu District, Guangzhou city of China, in August and September 2023. A stratified random sampling approach was used to select study districts. The 11 districts of Guangzhou were classified as urban or rural based on the dominant type of primary health institutions (e.g., community health centers vs. township hospitals) operating within their administrative boundaries. Panyu District, an economically developed and highly urbanized area, and Zengcheng District, a suburban–rural transitional district, were randomly selected to represent urban and rural regions of Guangzhou, respectively.

All FDTs across 32 primary health institutions in the two districts were enrolled in the survey. Eligibility criteria included: (1) over 6 months’ operational duration; (2) capacity and availability to complete the survey; (3) provision of informed consent; (4) participation of both team leader and at least two other team members. Ultimately, 224 FDTs met these criteria and were analyzed, achieving an 80.58% inclusion rate. Table 1 showed characteristics of the teams.

**Table 1:** Characteristics of the FDTs (n = 224).


VARIABLE	DESCRIPTIVE STATISTIC N (%)/MEAN (SD)

Team composition	

Basic teams (general practitioners, nurses and public health workers)	39 (17.41%)

Standard teams (general practitioners, nurses, public health workers and village doctors or specialists)	122 (54.46%)

Advanced teams (general practitioners, nurses, public health workers, village doctors or specialists and others)	63 (28.13%)

Team size	9.49 (3.85)

Establishment year	5.37 (2.97)

The ratio of contracted residents to general practitioners	2269.52 (2159.99)

IPC	3.97 (0.35)

Shared goals and visions	0.53 (0.07)

Internalization	0.63 (0.08)

Formalization	0.50 (0.08)

Governance	1.11 (0.14)

Incentive	1.19 (0.18)

IC	71.76 (8.00)

Professional integration	15.45 (6.21)

Cultural competence	21.48 (1.93)

Technical competence	34.82 (3.01)


According to Fritz and MacKinnon [16], a sample size of approximately 148 is required to detect small-to-medium mediation effects using bias-corrected bootstrap procedures (α = 0.05, power = 0.80). Our sample size of 224 exceeded this requirement.

### Data collection

Data were collected through questionnaires comprising three sections. The first section captured organizational demographics, including team size, establishment year, team composition, and contracted residents’ enrollment size. Regarding team composition, basic teams were defined as those including only general practitioners, nurses, and public health physicians. Standard teams were defined by the addition of specialists or village doctors to the core roles. Advanced teams were defined as standard teams further integrated with additional professionals, such as TCM practitioners, TCM pharmacists, and rehabilitation therapists.

The second section employed a structured evaluation instrument to assess IPC levels within FDTs. This instrument was developed based on a previously established IPC evaluation index framework [17]. The framework encompasses five dimensions, namely shared goals and visions [15], internalization [15], formalization [18], governance [19], and incentives [20]. Each item was rated on a 5-point Likert scale. Dimension scores were calculated by summing the item scores within each dimension. Team-level IPC scores were computed as the aggregate of dimension scores. This evaluation index framework was methodologically constructed and preliminarily validated in our prior research [17]. In this study, the instrument had a Cronbach’s α of 0.688. For structural validity, exploratory factor analysis showed KMO = 0.723 (Bartlett’s test of sphericity, *p* < 0.001), most item factor loadings were ≥ 0.40.

The third section measured the level of IC, using a validated Chinese version of the staff IC measurement tool [21]. The IC measurement tool includes three dimensions: professional integration [22], cultural competence [23] and technical competence [24]. These dimensions are assessed via a 5-point Likert-type scale ranging from “strongly disagree” to “strongly agree”. Dimension scores were calculated by summing the items within each dimension. Team-level IC scores were derived from the average of individual member scores. In this study, the scale had a Cronbach’s α of 0.960. For structural validity, exploratory factor analysis showed KMO = 0.956 (Bartlett’s test of sphericity, *p* < 0.001), and all item factor loadings were ≥ 0.40.

### Data analysis

Characteristics of the FDTs were described as the ordinal categorical variable (Basic/Standard/Advanced). Spearman correlation analysis was analyzed for the relationship between team composition, IPC, and IC. Before performing regression and mediation analyses, multicollinearity among independent variables was assessed by calculating variance inflation factors (VIFs) using multiple linear regression models [25]. Covariates with a VIF greater than 10 were considered to exhibit multicollinearity (see Appendix 1).

According to the method suggested by Professor VanderWeele [26], mediation analysis was conducted to examine whether IPC mediated the relationship between team composition and IC. Team composition was treated as a multicategorical variable and entered into the models using indicator (dummy) coding. Regression models were fitted to estimate the total effect of team composition on IC, the association between team composition and IPC, and the direct effect of team composition on IC after incorporating IPC into the model. All models adjusted for potential confounders, including team size, establishment year, and the ratio of contracted residents to general practitioners. Indirect effects were estimated using bias-corrected bootstrap procedures with 5,000 resamples. Mediation was considered statistically significant when the 95% bootstrap confidence interval excluded zero. The proportion mediated was calculated as the ratio of the indirect effect to the total effect. The proportion mediated was interpreted cautiously when the total effect was not statistically significant. All analyses were conducted using the PROCESS macro for SPSS 27.0.

To further examine the structural associations between the five dimensions of IPC and the three dimensions of IC, path analysis was performed using Stata 17.0. Model parameters were estimated via the maximum likelihood estimation method. The goodness-of-fit for the hypothesized models was assessed using the following indices: χ²/df < 3, Root Mean Square Error of Approximation (RMSEA < 0.08), Standardized Root Mean Square Residual (SRMR < 0.08), Goodness-of-fit Index (GFI > 0.90), Tucker–Lewis Index (TLI > 0.90), and Comparative Fit Index (CF > 0.90). All tests were two-tailed, with statistical significance set at *p* < 0.05.

### Ethics approval

This study was approved by the Biomedical Research Ethics Review Committee of the School of Public Health, Sun Yat-sen University (Approval No. [2022] 030). Written informed consent was obtained from all participants, and the whole study process followed the Helsinki Declaration.

## Result

### Characteristics for the FDTs

As shown in Table 1, a total of 224 FDTs were included in the study, of which 54.46% were standard teams, followed by advanced teams (28.13%), and basic teams (17.41%). The average establishment years of the teams was 5.37, and the mean ratio of contracted residents to general practitioners was 2269.52. The overall average score for IPC across all teams was 3.97, with higher scores in the incentive (1.19) and governance (1.11) dimensions, and lower scores in the dimensions of internalization (0.63), shared goals and visions (0.53), and formalization (0.50). For IC, the overall average score across the teams was 71.76, with the highest scores in the dimensions of technical competence (34.82), followed by cultural competence (21.48), and professional integration (15.45).

### Correlation analysis of team composition, IPC and IC

As shown in Table 2, there were significant correlations between team composition and IC, and between IPC and IC (*p* < 0.001). However, the correlation between team composition and IPC was not statistically significant (*p* = 0.124).

**Table 2:** Correlation analysis of team composition, IPC and IC.


VARIABLE	RELEVANCE (r)		

	TEAM COMPOSITION	IPC	IC

Team composition	1.000	–	–

IPC	0.103	1.000	–

IC	0.334***	0.503***	1.000


*Note:* ***p < 0.001.

### Mediation analysis of the association between team composition and IC via IPC

**Figure 1:**
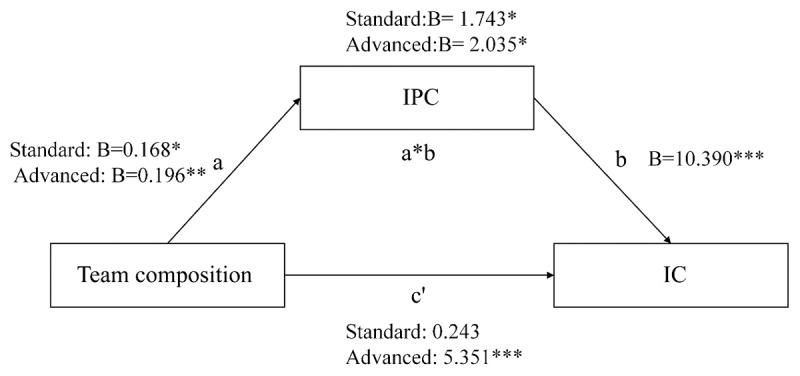
Mediation model of IPC in the association between team composition and IC. *Note*: *p < 0.05, **p < 0.01 and ***p < 0.001.

Table 3 shows the mediation analysis exploring whether IPC statistically mediates the association between team composition and IC. For standard teams compared with basic teams, the total effect on IC was not statistically significant (B = 1.986, 95% CI: –0.719 to 4.691, *p* = 0.149). However, the indirect effect via IPC was significant (B = 1.743, 95% CI: 0.490 to 3.128), while the direct effect was not significant (B = 0.243, 95% CI: –2.157 to 2.643, *p* = 0.842). For advanced teams compared with basic teams, the total effect was statistically significant (B = 7.386, 95% CI: 4.316 to 10.456, *p* < 0.001). Both the direct effect (B = 5.351, 95% CI: 2.624 to 8.077, *p* < 0.001) and the indirect effect via IPC (B = 2.035, 95% CI: 0.548 to 3.492) were significant. The indirect pathway accounted for approximately 27.55% of the total effect. The mediation model is presented in Figure 1.

**Table 3:** Mediation analysis of IPC in the association between team composition and IC.


COMPARISON	PATH	ESTIMATE (B)	95%CI	*P*-value

Standard vs. Basic	Team composition → IPC	0.168	0.040 to 0.295	0.010

	IPC→IC	10.390	7.916 to 12.864	<0.001

	Direct Effect	0.243	–2.157 to 2.643	0.842

	Indirect Effect	1.743	0.490 to 3.128	–

	Total Effect	1.986	–0.719 to 4.691	0.149

Advanced vs. Basic	Team composition → IPC	0.196	0.051 to 0.341	0.008

	IPC→IC	10.390	7.916 to 12.864	<0.001

	Direct Effect	5.351	2.624 to 8.077	<0.001

	Indirect Effect	2.035	0.548 to 3.492	–

	Total Effect	7.386	4.316 to 10.456	<0.001


*Note*: Basic teams as the reference group. The dependent variable was IC scores, and the mediator was IPC scores. All analyses were adjusted for team size, establishment year, and the ratio of contracted residents to general practitioners. A 95% confidence interval (CI) excluding zero indicates statistically significant mediation.

### Path analysis of the association between IPC and IC

**Figure 2:**
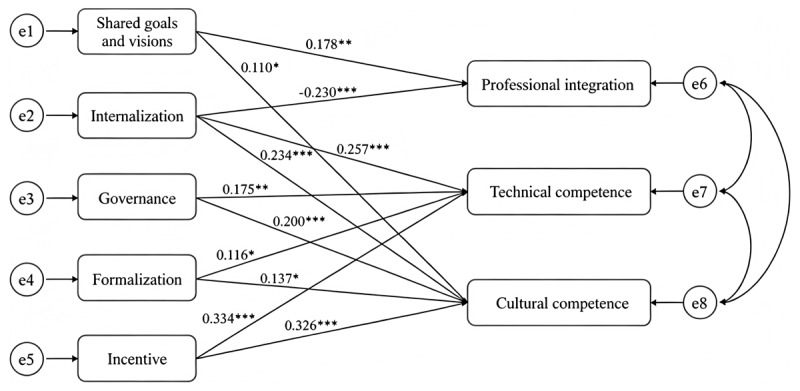
Path analysis of IPC effect on IC. *Note*: *p < 0.05, **p < 0.01 and ***p < 0.001.

Path analysis was conducted to examine the structural relationships between IPC and the multifactorial components of IC. The model fit indices were χ²/df = 2.47, GFI = 1.000, TLI = 0.993, RMSEA = 0.029, and SRMR = 0.011. As shown in Figure 2, shared goals and visions positively correlated with IC’s professional integration (B = 0.178) and cultural competence (B = 0.110). Internalization was negatively associated with professional integration (B = –0.230), while positively associated with technical competence (B = 0.257) and cultural competence (B = 0.234). Governance, formalization, and incentive were positively associated with both technical competence (B = 0.175, B = 0.116, B = 0.334) and cultural competence (B = 0.200, B = 0.137, B = 0.326).

## Discussion

This study is the first to empirically examine the association between team composition, IPC and IC within FDTs using urban-rural stratified sampling across China. It identified a potential mediating role of IPC and further explored pathways through which IPC was associated with IC, addressing the evidence gap on optimizing IC in FDTs.

### Discussion of the results

First, the non-significant bivariate correlation contrasts with the significant indirect effect observed in the mediation model. This discrepancy likely reflects the difference between simple unadjusted associations and conditional estimates derived from multivariable analysis.

Second, our findings indicate that the mediating mechanism linking team composition and IC differs by team type. In Standard teams, the association between team composition and IC was primarily observed through IPC, suggesting that collaborative processes may constitute the principal pathway through which compositional diversity relates to IC. In contrast, IPC only partially mediated this association in advanced teams, indicating the presence of additional pathways. Although prior studies have not directly tested IPC as a mediator between team composition and IC, evidence consistently shows that multidisciplinary teams and effective collaboration are associated with higher healthcare quality and better patient experiences [27,28]. Dai and colleagues noted that the effectiveness of team-based care depends on collaboration efficiency, irrespective of team composition [29]. Simply assembling professionals from diverse disciplines does not inherently ensure integrated care, as collaboration is inherently dynamic and complex. The differential pattern observed between advanced and standard teams suggests that greater compositional heterogeneity in advanced teams may operate through complementary mechanisms. Beyond IPC, structural features such as clearer role differentiation and more efficient task allocation may also be associated with IC [30], consistent with Bazemore et al.’s emphasis on multidisciplinary diversity in patient-centered care [31].

Finally, our study revealed a strong positive association between IPC and IC, and further path analysis identified shared goals and visions as most strongly associated with professional integration, consistent with He’s study [30]. This study also indicated that shared goals significantly influence the effectiveness of FDTs. Supper’s review identified shared goals and visions as facilitators of inter-professional teamwork in primary healthcare settings [32]. The primary purpose of teamwork is achieving team goals. Therefore, having specific, clear, and reasonable shared goals and visions may be important for enhancing team members’ motivation and forming a more collaborative professional team [33]. However, internalization was negatively correlated with professional integration. This may be associated with the current lack of effective inter-professional communication and internal conflict resolution mechanisms within Chinese family doctor contract system. Team members operate under internalized governance guidelines. However, absent such structured mechanisms, interpersonal interactions within teams may result in task boundary ambiguities, therefore undermining professional integration. Our study also found that incentive showed the strongest positive association with technical competence and cultural competence, consistent with previous studies [34]. Providing targeted spiritual and material incentives can meet care providers’ expectations and enhance intrinsic motivation for work, thereby stimulating cultural and technical competencies of the teams [30].

### Implications

Firstly, promoting diversity in team composition may help improve IC and IPC, based on the observed associations. To support this, team diversity could be considered in performance evaluation systems. This may encourage teams with basic compositions to recruit practitioners with complementary expertise. Secondly, team leaders could consider strengthening inter-professional coordination frameworks, which may be associated with more effective IPC. Equitable incentive allocation linked to collaborative achievements may help align individual rewards with collective outcomes, potentially fostering partnership synergies. Finally, standardizing inter-professional communication channels and establishing team mediation protocols could help address potential barriers to professional integration related to role internalization. These measures might be supported by education programs and mediation protocols.

### Limitation and further research

The limitations of this study should be acknowledged. First, the limited sample size restricted subgroup analyses based on team size and establishment year, limiting our understanding of inter-team differences. Future research could conduct subgroup analyses to explore how team characteristics affect team dynamics and outcomes. Moreover, the cross-sectional design of this study precludes causal inferences. Future studies could adopt longitudinal designs to examine the associations observed in this study and better assess potential causal relationships.

## Conclusion

The empirical study indicated that in advanced teams, team composition was positively associated with IC, with IPC partially mediating this relationship, whereas in standard teams, only the indirect effect via IPC was significant. Furthermore, the study showed that shared goals and visions were most strongly associated with professional integration, while incentives were most strongly associated with technical competence and cultural competence. These findings may inform strategies for optimizing integrated care, including promoting team diversity, strengthening collaborative governance, and establishing institutional mediation frameworks. All of these could support more effective care coordination.

## Appendix 1 Results of collinearity analysis

**Table S1:** Collinearity of independent variables in the regression analysis of team composition and IC.

	VARIANCE INFLATION FACTOR

Team composition	1.115

Team size	1.067

Establishment year	1.037

The ratio of contracted residents to general practitioners	1.034

**Table S2:** Collinearity of independent variables in the regression analysis of team composition and IPC.

	VARIANCE INFLATION FACTOR

Team composition	1.115

Team size	1.067

Establishment year	1.037

The ratio of contracted residents to general practitioners	1.034

**Table S3:** Collinearity of independent variables in the regression analysis of IPC and IC.

	VARIANCE INFLATION FACTOR

IPC	1.049

Team size	1.027

Establishment year	1.024

The ratio of contracted residents to general practitioners	1.020

**Table S4:** Collinearity of independent variables in the regression analysis of team composition, IPC and IC.

	VARIANCE INFLATION FACTOR

Team composition	1.145

IPC	1.077

Team size	1.107

Establishment year	1.062

The ratio of contracted residents to general practitioners	1.039
